# MBTI Personality Types of Korean Cabin Crew in Middle Eastern Airlines, and Their Associations with Cross-Cultural Adjustment Competency, Occupational Competency, Coping Competency, Mental Health, and Turnover Intention

**DOI:** 10.3390/ijerph18073419

**Published:** 2021-03-25

**Authors:** Mina Song, Hyun-jun Choi, Sunghyup Sean Hyun

**Affiliations:** 1 School of Tourism, Hanyang University, 222 Wangsimni-ro, Seongdong-gu, Seoul 04763, Korea; ssongmina@hanyang.ac.kr; 2 Department of Hotel and Foodservice Management, Cheng Ju University, 298 Daesung-ro, Cheongwon-gu, Cheong Ju 28503, Korea; chjun92@naver.com

**Keywords:** MBTI, personality, airline, cabin crew, flight attendant, cross-cultural adjustment competency, occupational competency, job competency, coping, stress coping, coping competence, mental health, turnover intention

## Abstract

The purposes of this study were (1) to identify MBTI (Myers–Briggs Type Indicator) personality profiles of Korean cabin crew in Middle Eastern airlines, (2) to determine whether MBTI personality affects their cross-cultural adjustment competency, occupational competency, and coping competency, and (3) to analyze the impact of these variables on their mental health and turnover intention. Furthermore, we verified (4) the moderating effect of cabin crew’s previous overseas experience on the relationship between cross-cultural adjustment competency and turnover intention. MBTI-Form M test and a survey questionnaire were distributed to 185 Korean cabin crew members in Middle Eastern airlines, and 172 valid datapoints were used for analysis. It was revealed that the cabin crew members showed significantly different levels of cross-cultural adjustment competency, occupational competency, and coping competency depending on their personality traits. Furthermore, those with higher cross-cultural adjustment competency and stress coping are more likely to have positive mental health, which also had an influence on lowering their turnover intention. Occupational competency had no significant association with mental health; however, it directly affects turnover intention. The findings will contribute not only to career plan guidelines for cabin crew aspirants, but also to airlines’ recruitment strategies as well as human resources management in aviation industry.

## 1. Introduction

In today’s competitive market environment in the aviation industry, cabin crew are considered important assets for success [1]. Although cabin crew are important front-line employees in the aviation industry, they often encounter hassles in their work due to the pressure of operating safety duties and providing outstanding service at the same time [2]. To meet the standards of proficiency, a variety of qualifications and competencies are required such as a sociable personality, positive attitude, empathy, and decision-making skills. In addition, the cabin crew must constantly make efforts to keep customer satisfaction at a high level, which includes resolving complaints.

Meanwhile, the aviation industry in the Middle East has expanded rapidly over the past decade, establishing itself as the fastest-growing, winning numerous international awards. As multicultural companies, they recruit cabin crew from more than 100 different countries, regardless of nationality, race, religion, or gender. Upon employment, the cabin crew are relocated to the Middle East, where they experience a completely different culture from their homeland. For this reason, airlines look for applicants who are open-minded to other cultures and have the ability to adapt to new environments without difficulty. Nevertheless, cabin crew in Middle Eastern airlines have shown higher rates of resignation than other domestic airlines. Recent research has reported that the main causes of high turnover rates for foreign cabin crew are, first, acculturation stress, including culture shock and homesickness; and second, job stress caused by emotional exhaustion, jet lag, shift work, etc. [3,4,5,6,7].

In this study, we focused on personality as the crucial factor influencing cabin crew members’ competency such as cross-cultural adjustment competency, occupational competency, and coping competency. According to the previous studies, there is a particularly strong correlation between personality and cross-cultural adjustment [8,9,10,11,12]. It has been even suggested that personality is one of the more important factors to help for effective adjustment than professional work skill, which can be learned through special training [13]. Furthermore, it has been proven by earlier studies that personality is an important factor to predict individual’s job performance or work-related competency [14,15,16].

We used the Myers–Briggs Type Indicator (MBTI) to identify the personality type of each respondent and divided them into groups to find out if MBTI profiles of Korean cabin crew in Middle Eastern airlines show different distribution compared to cabin crew in Korean domestic airlines, which appeared ESTJ (19.3%, *n* = 45) was the dominant types followed by ESFJ (15.9%, *n* = 37) and ESFP (12.9%, *n* = 30) [17]. We also tried to investigate whether cabin crew shows different levels of cross-cultural competency, occupational competency, and coping competency depending on their personality types, as well as to determine whether these variables can affect individual mental health and turnover intention.

## 2. Literature Review

### 2.1. Myers–Briggs Type Indicator (MBTI)

MBTI theory, inspired by Carl G. Jung’s theory of psychological types, classifies human personality into 16 different types on the basis of four dichotomies: extraversion/introversion, sensing/intuition, thinking/feeling, and judging/perceiving. Each preference pair has distinctive functions and characteristics.

Extraversion (E) types gain energy from being outside and mingling with people. They tend to bond easily with strangers, and enjoy talking with others almost without any difficulty. On the other hand, Introversion (I) types gain energy from their inner world of ideas and experiences; thus, they prefer to communicate in writing and process information at their own pace quietly. Sensing (S) types use their own senses to take in information. They are most concerned with the present and empirical evidence, and are often detail-oriented and rule-focused. In contrast, Intuition (N) types rely on insights and process information through a big picture or patterns, rather than details. They tend to enjoy designing new ideas and challenging the diversity of life. Thinking (T) types make decisions by rational, critical thinking based on objective criteria and utilize logical thought processes to solve problems. They are considered businesslike people, and focus on the result of the project, rather than the process or harmony. Contrariwise, feeling (F) types are concerned with personal relationships, and judge situations based on their feelings, personal values, and social considerations. They focus on emotional communications and are motivated by the desire to help others. Judging (J) types approach life in a structured and organized way. They like to set a detailed plan for their schedules, and follow it strictly. Similarly, they like to keep their areas neat and tidy as a daily routine. Opposite to Judging (J) types, Perceiving (P) types approach the life in a freewheeling and spontaneous way. They tend to perceive structure as restriction, and prefer flexibility [18,19,20,21].

### 2.2. Cross-Cultural Adjustment Competency

The term of adaptation or adjustment was originally conceptualized within evolutionary biology, referring to a group’s survival in a new physical environment. The definition has been expanded to the social and cultural spheres; it was interpreted in the context of the development of behavioral and psychological adaptative mechanisms [22]. Cross-cultural adjustment is often called acculturation, which refers to a process of social, psychological, and cultural change that stems from the balancing of two cultures while one adapts to the prevailing culture of the society [23]. In the cross-cultural experience, people readjust themselves to another culture. In this process, they may face changes in lifestyles and thinking principles that would lead them to acquire distinct changes in perception or physical/mental life [24]. However, environmental changes may create acculturation stress such as culture shock, homesickness, anxiety, loneliness, and depression [23,25,26]. Failure to recover from this stress can have a negative impact on an individual’s physical health as well as mental health [27,28,29], and it may cause premature returns. In this study, cross-cultural adjustment competency is defined as the ability of the cabin crew to accept and respect different cultures with open minds, and the capacity to successfully adjust to cultural changes.

In fact, cabin crew in the Middle East often experience adaptation difficulties upon relocating due to different cultural and language surroundings. Culture shock, homesickness, and miscommunications can occur—unavoidably—when working with foreign passengers and foreign colleagues, and when they immerse themselves in Arab culture. For these reasons, when Middle Eastern airlines recruit cabin crew, they clearly specify that they are seeking candidates who are open-minded to other cultures, with cultural awareness, and who have a positive personality with resilience.

Among the influential factors contributing to how individuals react to acculturation, many researchers have emphasized personality characteristics such as likeability, sociability, extraversion, and ego control [30,31]. According to some researchers, extroverts have the ability to establish friendships with host nationals, which consequently facilitates the social learning of culture-specific skills, helping cross-cultural adjustment [32,33,34]. There have also been many attempts to measure cross-cultural adjustment competency and personality using the Big Five personality theory. Additionally, it was demonstrated that the personality traits of extraversion, agreeableness, conscientiousness, and (less) neuroticism are significantly related to cross-cultural adjustment [12].

Based on this theoretical and empirical background, we investigated whether MBTI personality traits can be an influential factor determining individual competency to accept unfamiliar cultures without difficulties and to enjoy exploring the changes.

**H1.**  *MBTI personality types of cabin crew significantly influence cross-cultural adaptation competency.*

### 2.3. Occupational Competency

Occupational competency refers to the potential capacity of an individual to successfully handle certain situations or complete specific tasks [35]. Researchers in organizational psychology have argued that personality is the best predictor of human behavior and motivational influences on the individual’s work performance [36]. Due to the usefulness of personality predictors in determining individual job performance, there have been numerous attempts to explain the relationships between personality traits and occupational competence in various occupations, including students, teachers, and business executives [37,38,39,40]. This study aimed to investigate whether cabin crew showed different competencies toward their respective duties depending on personality types using MBTI theory.

Being part of a cabin crew is a profession that is responsible for the safety, security, and welfare of passengers from the time they board the aircraft to the time they disembark. Referring to the service and safety manuals from three major full-service Middle Eastern airlines—Emirates, Etihad Airways, Qatar Airways—we classified cabin crew duties and responsibilities along five dimensions: (1) Cabin Service Operating, (2) Galley Operating, (3) Duty Free Sales, (4) Safety Duties during Normal Operations, and (5) Safety Duties during Abnormal and Emergency Situations. After thorough reviewing the service and safety manuals of Middle Eastern Airlines, we describe the procedures regarding their duties and responsibilities as follows.

#### 2.3.1. Cabin Service Operating

In Middle Eastern airlines, all crew members except for onboard leaders are assigned specific positions and working areas for in-flight service duties, either as a cabin service operator or galley operator, and these positions change on every flight. The members assigned cabin service operator tasks have the responsibilities of welcoming passengers during boarding, serving onboard meals, approaching passengers for engagement, and resolving customer complaints; this means that cabin service operators have to directly interact with passengers by having face-to-face conversations.

#### 2.3.2. Galley Operating

The galley refers to the facility of an aircraft where food is cooked and prepared. This facility is composed not only of compartments to serve and store food and beverages, but also storage spaces for anything that the cabin crew might need during the flight and for emergencies. Before departure, the galley operators must carry out galley and catering checks to ensure that equipment such as trolleys and containers are securely stowed and that all catering equipment such as ovens, boilers, chillers, and coffee makers are fully serviceable. They also must check whether the correct numbers of service items and passenger meals are loaded in the correct locations. During the cruise, they heat the foods and set up the carts, acting as supporters to deliver the service in a timely manner. At the same time, they make sure to organize and clean the galley area. To perform these duties successfully, galley operators need organizational skills and the ability to prioritize tasks as well as count items with accuracy.

#### 2.3.3. Duty Free Sales

Cabin crew are also responsible for promoting duty free products and conducting retail sales during flights. Cabin crew must be aware of detailed product information, and they need to proactively interact with the passenger by recommending the products in accordance with personal needs and preferences.

#### 2.3.4. Safety Duties during Normal Operations

Occupational competency for safety duties as a cabin crew can be referred to as safety behaviors. On every flight, the cabin crew must ensure that all aspects of safety and security duties are completed during the different stages of the flight. For instance, during pre-passenger boarding, they carry out safety and security checks of the cabin, emergency equipment, lavatories, overhead stowage, and passenger seats thoroughly in their assigned area of responsibilities. Safety duties during normal operations are conducted on a daily basis in order to identify hazards and prevent potential hazards that may endanger or affect flight safety. In order to accomplish these duties, cabin crew must be familiar with every safety procedure and comply with the aviation safety regulations and company policy, as well as update bulletins and safety highlights before flight.

#### 2.3.5. Safety Duties during Abnormal and Emergency Situations

The main duty of the cabin crew is to ensure the safety of the aircraft and passengers, and this is especially crucial in emergency situations. Emergency situations that may occur in the aircraft include emergency evacuation, decompression, firefighting, medical emergencies, bomb threats, hijacking, and dealing with disruptive passengers. It is mandatory for cabin crew to attend annual Safety Procedures Training to be able to deal with any incidents onboard confidently [41] and attending safety training is essential to enhance safety knowledge and skills. However, individual safety behaviors or personality competences to deal with emergencies are also considered a key element, for example, decision-making skills, assertiveness, and the ability to handle the situation calmly.

Based on previous studies, we assumed that cabin crew would show different abilities in terms of job performance, depending on individual personality traits. Therefore, the following hypothesis was proposed:

**H2.**  *The MBTI personality types of cabin crew members will significantly affect their individual occupational competency in their respective duties.*

### 2.4. Coping Competency

Previous research argued that cabin crew tend to endure more responsibility and pressure than other customer-service-related front-line employees in different industries, due to the additional responsibilities of caring for safety, security, and passenger services [42]. In addition, people in the cabin crew profession face many negative working conditions while performing their duties, such as working long hours in a confined space and being exposed to emotional exhaustion because of customers. These factors lead cabin crew to experience a high degree of distress and frustration [43]. Crew members in Middle Eastern airlines in particular face cumulative challenges caused by acculturation stress because they must live in their base city in a Middle Eastern country.

The term coping competence is generally defined as “the capacity to effectively cope with failure and negative life events as indicated by a reduced likelihood of helplessness reactions and fast recovery from any occurring helplessness symptoms [44].” Essentially, “the most significant feature of coping competency is a resistance against a depressogenic attributional style and the respective motivational deficits in times of stress and crisis [45].”

There is considerable evidence from earlier studies that MBTI personality traits can influence psychological stress. For instance, it was reported that among a population of unipolar depressed patients, 74% of them were classified as Introversion type, 75% as Sensing type, and 84% as Feeling type [46]. It was also found that a significant correlation exists between Extraversion personality and ego-resilience as well as self-efficacy among college students [47]. In addition, Introverted students in nursing colleges tend to perceive stress as greater than Extraverted students do. Extraversion type students adapt themselves to school better than Introversion types, and their level of stress coping is higher [48]. Accordingly, the following hypothesis is proposed.

**H3.**  *MBTI personality types of cabin crew members will significantly influence their coping competency.*

### 2.5. Mental Health and Turnover Intention

According to the World Health Organization, mental health is “a state of well-being in which the individual realizes his or her own abilities, can cope with the normal stresses of life, can work productively and fruitfully, and is able to make a contribution to his or her community” [49]. Mental health and mental hygiene are also defined as “a condition, subject to fluctuations due to biological and social factors, which enables the individual to achieve a satisfactory synthesis of his own potentially conflicting, instinctive drives; to form and maintain harmonious relations with others; and to participate in constructive changes in his social and physical environment” [50].

Although there are various criteria for measuring mental health, many researchers suggest depression as the most relevant indicator [51]. Studies of depression conducted by Beck suggest that depressed people tend to have a negative view of the outside world and their future, which can lead to negative cognitive distortion, sadness, passivity, self-criticism, and even suicidal ideation [52].

In terms of the associations of cross-cultural adjustment and mental health, cultural differences can cause communication barriers, misunderstandings, and even conflicts between groups that have disparate cultural values. The inability to handle cultural differences and adjust to a new cultural environment can have a negative impact on individual well-being that will eventually result in burnout [53]. Back et al. argued that acculturative stress factors, including homesickness, culture shock, and fear, were associated with depression in Korean cabin crew residing overseas [6]. Furthermore, it was confirmed that acculturation stress and depression lead to cabin crew turnover intention, which refers to the employees’ behavioral intention and perceived probability of leaving the current organization [54]. Similarly, Kim et al. confirmed that perceived discrimination, homesickness, and sense of inferiority as acculturative stress factors were associated with burnout in Korean cabin crew working in Middle Eastern airlines [3].

In the area of work and occupational psychology, there have been many studies of work stressors and their associations with mental, physical, and behavioral stress reactions, such as depression, burnout, and psychosomatic diseases [55,56,57]. Acker and Lawrence highlighted that social workers who perceived competency in their abilities showed lower levels of role stress and burnout [58]. It was also found that job demand significantly affected burnout among Taiwanese cabin crew. Moreover, a high level of burnout aggravated cabin crew’s health problems, which directly affected turnover intention [43]. Based on the discussion above, the following hypotheses are proposed:

**H4.**  *A high level of cross-cultural competency will positively and significantly influence mental health.*

**H5.**  *A high level of occupational competency will positively and significantly influence mental health.*

**H6.**  *A high level of coping competency will positively and significantly influence mental health.*

**H7.**  *A high level of cross-cultural adjustment competency will positively and significantly influence turnover intention.*

**H8.**  *A high level of occupational competency will negatively and significantly influence turnover intention.*

**H9.**  *A high level of coping competency will negatively and significantly influence turnover intention.*

**H10.**  *A high level of mental health will negatively and significantly influence turnover intention.*

### 2.6. Moderating Effect of Overseas Experience on the Relationship between Cross-Cultural Adjustment Competency and Turnover Intention

A team of researchers in Korea conducted a study of stress in cabin crew in Middle Eastern airlines. They found that among the variables of acculturation stress, perceived discrimination, homesickness, and inferiority presented differently, depending on whether they had experience living abroad before joining the airlines [3]. In other words, the group without overseas experiences tended to perceive more stress while they adapted to Middle Eastern culture than those who had lived in other countries for study or work.

In fact, when airlines recruit crew members, they consider overseas experience an important qualification of candidates, because they assume that a candidate with overseas experience tends to be more open-minded, respects other cultures, and flexibly handles difficult situations in the process of acculturation, since they have already had experience being relocated, expecting that they thus know how to successfully manage acculturation stress.

**Figure 1:**
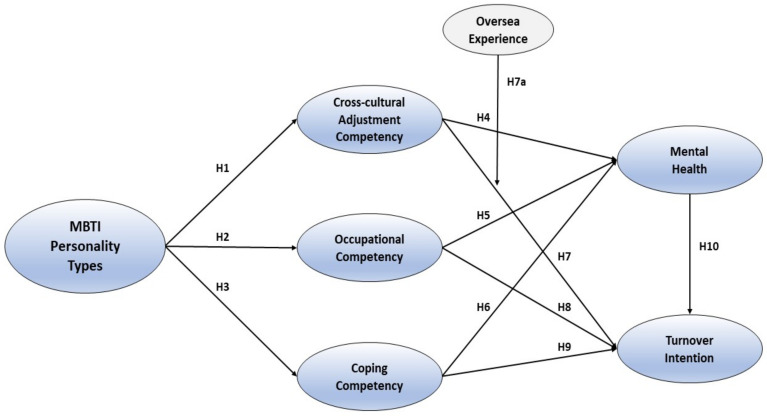
Conceptual model.

Based on previous research results, we sought to determine whether cabin crew members’ previous overseas experience plays an important role in the relationship between cross-cultural adjustment competency and turnover intention. Figure 1 provides the potential study model of this research.

**H7a.**  *The presence of overseas experience will have a moderating effect on the relationship between cross-cultural adjustment competency and turnover intention.*

## 3. Methods

### 3.1. Study Design and Participants

A cross-sectional descriptive analysis was conducted using the Korean version of the MBTI-Form M tool and a separate survey questionnaire. The criteria of samples should be (1) Korean native speakers with Korean nationality to fully understand questionnaire, and (2) cabin crew members with at least six months of work experience in three major full service Middle Eastern airlines were invited. The sample group was limited to the cabin crew in full-service airlines because they have similar flight operation sequences in terms of duties for cabin service, galley operating, duty free sales, etc. Additionally, they have similar procedures for assigning cabin crew positions onboard. We advertised for research volunteers on the Korean cabin crew community website. All volunteers were informed that the data will be kept confidential and destroyed after the analysis is completed. After obtaining consent to participate in this research, the volunteers received invitations with MBTI access serial numbers via email. Once participants completed the online MBTI self-report test, they received the test results along with the questionnaires for evaluating their level of cross-cultural competency, occupational competency, coping competency, mental health, and turnover intention. A total of 185 personality tests and surveys were distributed from 3 August to 9 October 2020. However, 2 participants refused to conduct the personality test due to concern of leaking personal information, 8 participants did not finish the test, and 3 missing datapoints were found. Therefore, the data of the 172 subjects deemed valid were used for statistical analysis.

### 3.2. Measures

To empirically measure the 10 theoretical concepts proposed in this study, validated measurement items were adapted from the existing literature in various areas (psychology, cabin crew competency, customer orientation, etc.) as shown below:

- Cross-cultural competency was measured with 4 items based on open-mindedness, flexibility, emotional stability, and social initiative from the MPQ (Multicultural Personality Questionnaire) [59,60].
- Occupational competency was measured with 16 items under 5 dimensions of cabin crew duties: (1) cabin service operating, (2) galley operating, (3) duty free sales, (4) safety duties during normal operations, and (5) safety duties during abnormal and emergency situations. The questions were adapted from cabin crew job competency scale by Park [61], Lee [62], a new scale of social desirability independent of psychopathology by Crown and Marlow [63], a measure of the customer orientation of salespeople by Saxe and Weitz [64], and safety behaviors scales by Chen and Chen [65], Kim [66], Seo [67], and Kwon [68].
- Coping competency was measured with 3 items employed from The Brief Resilience Scale: Assessing the ability to bounce back by Smith [69].
- Mental health was measured with 5 items adapted from Symptom Check List-90-Revision: SCL-90-R by Kim, Kim, and Won [70,71].
- Turnover intention was measured with 3 items adapted from turnover intention scale by Lawler [72].

The initial questionnaire was created by combining the measurement items named above (all measurement items in this study, except for galley operating) and had the subject evaluate them on a five-point Likert scale from 1 (“strongly disagree”) to 5 (“strongly agree”). To ensure the content validity of the initial questionnaire, we had a focus group interview with current cabin crew in Middle Eastern airlines and other professional groups. We thoroughly reviewed all items, and the initial questionnaire was revised based on their feedback. A pre-test was then conducted with 20 cabin crew members to confirm readability. Cronbach’s alpha values higher than 0.7 further supported the reliability of the adapted scales.

### 3.3. Data Analysis

T-test was performed to examine the differences in cross-cultural adjustment competency, occupational competency, and coping competency, depending on MBTI personality traits (E-I, S-N, T-F, J-P). The descriptive statistics of all scales and measures for variables were assessed.

The bivariate correlations among cross-cultural adjustment competency, occupational competency, coping competency, mental health, and turnover intention were analyzed by Pearson’s correlation coefficients.

Hierarchical multiple linear regression analysis following Baron and Kenny [73] was used to measure the effects of cross-cultural adjustment competency, occupational competency, and coping competency on mental health and turnover intention.

Multiple regression analysis was performed to verify the moderating effect of overseas experience in the relationship between cross-cultural adjustment competency and turnover intention.

## 4. Results

### 4.1. Demographic Profile of the Respondents

We collected data fairly evenly from three airlines: Emirates (29.7%, *n* = 51), Etihad Airways (33.1%, *n* = 57), and Qatar Airways (37.2%, *n* = 64). Among the 172 survey participants, about 97.7% (*n* = 168) were female and 2.3% (*n* = 4) were male. Regarding education level, about 84.3% (*n* = 145) of the respondents had a bachelor’s degree, followed by 2-year college degrees (9.9%, *n* = 17), and graduate degrees (5.9%, *n* = 10). There were no respondents who only had a high-school education.

The subjects who were 30–34 years old made up the largest portion (48.8%, *n* = 84), followed by 35–39 (25.0%, *n* = 43), 25–29 (18.0%, *n* = 31), and 40 and above (8.1%, *n* = 14). Regarding marital status, 69.2% (*n* = 119) were single and 30.8% (*n* = 53) were married. The survey participants were asked to indicate their position. Most respondents were Economy Class crew (45.9%, *n* = 79), followed by Business Class crew (30.2%, *n* = 52), First Class crew (12.2%, *n* = 21), and Onboard Leader (11.6%, *n* = 20). Regarding work experience, 28.5% (*n* = 49) of the respondents had work experience between 5 and 8 years, 27.9% (*n* = 48) had experience between 1 and 3 years, 26.7% (*n* = 46) had experience between 3 and 5 years, those with more than 8 years made up 9.9% (*n* = 17), and those with less than 1 year made up 7.0% (*n* = 12). Lastly, 75.6% (*n* = 130) of respondents answered that they had had previous overseas experience before joining the airlines.

### 4.2. MBTI Personality Types of the Respondents

Table 1 shows the distribution of MBTI types of the respondents. The most dominant MBTI personality type among Korean cabin crew members in the Middle Eastern airlines was ENFP (15.1%, *n* = 26), followed by ESTJ (14.0%, *n* = 24) and ESFJ (10.5%, *n* = 18). In terms of individual traits, Extroversion was exhibited by 114 (66.3%), Sensing traits by 113 (66.7%), Feeling traits by 97 (56.4%), and Judging traits by 88 (51.2%).

### 4.3. MBTI Personality Types and Cross-Cultural Adjustment Competency

We used an independent *t*-test to identify whether personality, as measured by the Myers–Briggs Type Indicator, affects the level of cross-cultural adjustment competency, occupational competency, and coping competency. Extroversion (E), Intuition (N), and Perceiving (P) types tend to be more open-minded to cultural differences, adjust themselves better to the new cultural environment, and enjoy participating in social gatherings with foreigners. There was no significant difference between Thinking (T) and Feeling (F) traits.

### 4.4. MBTI Personality Types and Occupational Competency

Cabin crew duties were divided into five dimensions: (1) cabin service operating, (2) galley operating, (3) duty free sales, (4) safety duties during normal operations, and (5) safety duties during abnormal operations. The results of the analysis indicate that Extroversion (E), Intuition (N), and Feeling (F) types demonstrate outstanding abilities in cabin service operating. They enjoy approaching passengers for engagement and confidently handle complaints with empathy in comparison to Introversion (I), Sensing (S), and Thinking (T) types.

On the other hand, Sensing (S), Thinking (T), and Judging (J) types exhibit greater competence in galley operating duties. They tend to show accuracy in counting the number of onboard meals/service items, and in keeping the galley clean and organized during the flight.

Extroversion (E), Intuitive (N), and Feeling (F) types exhibit excellent selling skills when carrying out duty free sales. They tend to approach the passengers proactively for duty free sales to recommend the products according to passengers’ needs/preferences, and to deliver correct information about the products.

Interestingly, no significant differences were found by personality type in conducting safety duties during normal operations. To find out specific implications of this, we conducted in-depth interviews with respondents. Cabin crew carry out these tasks on a regular basis, and they tend to devote extra care to these duties, considering that even a small mistake on these duties can lead to serious issues onboard. All personality types tend to present different strengths and weaknesses toward certain situations. Even if the cabin crew might make mistakes when off duty, they try to overcome their weaknesses and act with particular care when conducting safety duties.

For handling safety duties during abnormal operations—meaning emergency situations such as firefighting, decompression, and handling disruptive passengers—cabin crew are required to be prompt, alert, and assertive, as well as possess great decision-making skills. Table 2 indicates that Extroversion (E) and Thinking (T) types show greater competency levels for handing emergency situations than Introversion (I) and Feeling (F).

### 4.5. MBTI Personality Types and Coping Competency

For coping competency, it was found that the Extroversion (E) types show a higher level of coping competency than the Introversion (I) types. The cabin crew members with an outgoing and extroversive personality tend to have an outstanding ability to overcome difficult situations with ease and move on quickly from setbacks.

### 4.6. Correlation Analysis

A Pearson correlation analysis was performed to identify relationships among the variables. The results are shown below in Table 3.

There was a positive correlation between cross-cultural adjustment competency and occupational competency (*r* = 0.439, *p* < 0.001). Among the duties of occupational competency, cross-cultural adjustment competency showed a positive correlation with cabin service operation (*r* = 0.549, *p* < 0.001), duty free sales (*r* = 0.437, *p* < 0.001), and safety duties during abnormal operation (*r* = 0.342, *p* < 0.001). Cross-cultural competency also showed a positive correlation with coping competency (*r* = 0.455, *p* < 0.001) and mental health (*r* = 0.397, *p* < 0.001); however, a negative correlation was observed between cross-cultural adjustment competency and turnover intention (*r* = 0.390, *p* < 0.001).

Occupational competency showed a positive correlation with coping competency (*r* = 0.235, *p* < 0.01), and a negative correlation with turnover intention (*r* = −0.302, *p* < 0.001). Among the dimensions of occupational competency, cabin service operating (*r* = 0.202, *p* < 0.01), duty free sales (*r* = 0.275, *p* < 0.001), and safety duty during abnormal operation (*r* = 0.257, *p* < 0.001) showed a positive correlation with coping competency. Cabin service operating (*r* = 0.271, *p* < 0.001), duty free sales (*r* = −0.249, *p* < 0.01), and safety duty during abnormal operation (*r* = −0.218, *p* < 0.01) had a negative correlation with turnover intention, and safety duty during normal operation had a negative correlation only with turnover intention (*r* = −0.263, *p* < 0.001).

Coping competency showed a positive correlation with mental health (*r* = 0.540, *p* < 0.001), but a negative correlation with turnover intention (*r* = −0.392, *p* < 0.001). Mental health had a negative correlation with turnover intention (*r* = 0.524, *p* < 0.001).

### 4.7. Hierarchical Multiple Linear Regression Analysis

Hierarchical multiple linear regression analysis was used to measure the effect of cross-cultural adjustment competency, occupational competency, and coping competency on mental health and turnover intention, in order to identify any mediating effect of mental health between these variables. Table 4 shows the analysis outputs.

First, the analysis of the effects of cross-cultural adjustment competency, occupational competency, and coping competency on mental health verified that a fit regression model exists (*F* = 26.025, *p* < 0.001), and the explanatory power of the regression model was approbatively 31.7%. The result of verifying the significance of the regression coefficient for cross-cultural adjustment competency (β = 0.196, *p* < 0.05) and coping competency (β = 0.466, *p* < 0.001) indicated a positive effect on mental health, which means that those with higher levels of cross-cultural adjustment competency and coping competency are more likely to present a positive mental health condition.

Second, the analysis of the effect of cross-cultural adjustment competency, occupational competency, and coping competency on turnover intention verified that a fit regression model exists (*F* = 16.567, *p* < 0.001), and the explanatory power of the regression model was approbatively 22.8%. The result of verifying the significance of the regression coefficient, cross-cultural adjustment competency (β = 0.204, *p* < 0.05), occupational competency (β = −0.150, *p* < 0.001), and coping competency (β = −0.264, *p* < 0.01) indicated a negative effect on turnover intention. It was also shown that mental health has a negative effect on turnover intention (β = 0.416, *p* < 0.001), indicating that cross−cultural adjustment competency and coping competency affect cabin crew members’ intention to leave the company through the mediating effect of mental health. Additionally, it was verified that occupational competency directly had a negative effect on turnover intention (β = −0.177, *p* < 0.05). In other words, the cabin crew members with higher mental health levels showed a lower level of turnover intention.

### 4.8. The Moderating Effect of Overseas Experience

In this study, 75.6% (*n* = 130) of respondents had experience living in another country for at least six months, while 24.4% (*n* = 42) had no overseas experience. We conducted a multiple regression analysis to verify the moderating effect of overseas experience on the relationship between cross-cultural adjustment competency and turnover intention by separating the two sample groups. Overall, a high level of cross-cultural adjustment competency negatively affected turnover intention. However, Table 5 shows that cross-cultural adjustment competency for cabin crew members without overseas experience did not influence turnover intention (β = 0.056, t = 0.379, *p* < 0.707). On the other hand, cross-cultural adjustment competency for cabin crew members with overseas experience significantly influenced turnover intention (β = −0.258, t = −0.753, *p* < 0.007), implying that previous overseas experience has a positive effect on adjusting to new cultural environments, as well as lessening the desire to leave the occupation.

### 4.9. Hypothesis Testing

**Figure 2:**
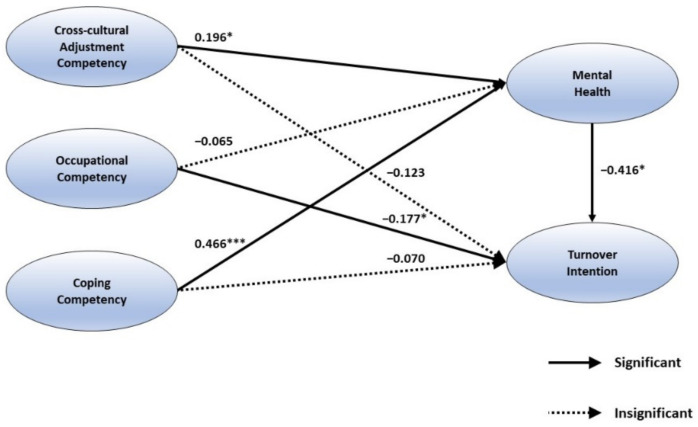
Structural model result. Note: * *p* < 0.05, *** *p* < 0.001.

Figure 2 provides the hypothesis testing results. The proposed impact of MBTI personality types on cross-cultural adjustment competency, occupational competency, and coping competency was tested (H1, H2, H3), finding that the cabin crew members showed different levels of cross-cultural adjustment competency, occupational competency, and coping competency depending on their MBTI personality types, which supports Hypotheses 1, 2, and 3. Hypotheses 4, 5, and 6 were also tested, finding a significant association between cross-cultural adjustment competency and mental health (β = 0.196, *p* < 0.012), and between coping competency and mental health (β = 0.466, *p* < 0.000). These results supported Hypotheses 4 and 6. On the other hand, occupational competency did not impact mental health (β = −0065, *p* < 0.358). Thus, Hypothesis 5 was not supported. Hypotheses 7, 8, 9, and 10 were then tested. Our findings showed that occupational competency directly influenced turnover intention (β = −0.177, *p* < 0.012); in addition, mental health also had a direct impact on turnover intention (β = −0.416, *p* < 0.000), which supported Hypotheses 8 and 10. Although cross-cultural adjustment competency and coping competency did not have a direct impact on turnover intention, they indirectly influenced turnover intention through the mediating effect of mental health. Hence, the results supported Hypotheses 7 and 9. The variables within the proposed theoretical framework explained 24.2% of the total variance in cross-cultural adjustment competency, occupational competency, coping competency, and mental health, and 22.8% of the variance in cross-cultural adjustment competency, occupational competency, coping competency, and turnover intention. Table 6 summarizes the hypothesis testing results.

## 5. Discussion

The aim of the present study was (1) to identify personality profiles of Korean cabin crew in Middle Eastern airlines using MBTI (Myers–Briggs Type Indicator), (2) to investigate the correlation between personality and their cross-cultural adjustment competency, occupational competency, coping competency, and (3) to analyze the impact of these variables on their mental health and turnover intention. In addition, we verified (4) whether the cabin crew’s previous overseas experience has a moderating effect on the relationship between cross-cultural adjustment competency and turnover intention.

First, this study demonstrated that cabin crew in Middle Eastern airlines show a demographically distinctive distribution of traits in their MBTI personality profile, compared to cabin crew in Korean domestic airlines as well as employees in other industries. According to the result of data analysis, the most dominant personality types of among Korean cabin crew in the Middle Eastern airlines was ENFP (15.1%, *n* = 26), followed by ESTJ (14.0%, *n* = 24) and ESFJ (10.5%, *n* = 18), whereas regarding the MBTI analysis of Korean domestic airlines cabin crew showed that ESTJ (19.3%, *n* = 45) was the most common type, followed by ESFJ (15.9%, *n* = 37) and ESFP (12.9%, *n* = 30). Meanwhile, the least common types were INTJ (1.2%, *n* = 2), ENTP (1.7%, *n* = 3), INTP (1.7, *n* = 3), and ENTJ (2.3%, *n* = 4). This distribution exhibited similar results with Korean domestic airlines cabin crew (INTP: 0%; ENTP: 0.9%, *n* = 2; INTJ: 1.3%, *n* = 3; ENTJ: 1.3%, *n* = 3) [17]. In terms of individual indexes, Extroversion (E) accounted for 66.3% (*n* = 114), while Introversion (I) was 33.7% (*n* = 58). This result reflects the fact that the cabin crew recruitment process generally involves public speaking interview, and many airlines remind in job descriptions that they look for candidates who are sociable and outgoing to enjoyably engage with passengers onboard. Sensing (S) types were 65.7% (*n* = 113); on the other hand, Intuition (N) types were 34.3% (*n* = 59). While Feeling (F) types accounted for 56.4% (*n* = 97), Thinking (T) types were 43.6% (*n* = 75). Cabin crew in Korean airlines also showed a similar outcome (Feeling: 62.2%, *n* = 145; Thinking: 37.8%, *n* = 88). It demonstrated that regardless of cultures and base of companies, for important personality as cabin crew, airlines generally show more concern for empathy, consideration, and desire to help others with a caring attitude than being rational and logical. Lastly, there was no significant differences between Judging types (51.2%, *n* = 88) and Perceiving types (48.8%, *n* = 84).

Second, it was revealed that an individual’s personality had a significant effect on cross-cultural adjustment competency, which can also impact mental health and intention to resign. This finding corresponds to previous studies highlighting that personality is an essential variable affecting an individual’s ability in terms of cultural adaptation [8,9,10,11,12,13], as well as research which verified that the competence of individuals in functioning effectively in cross-cultural situations plays an important role in lowering stress and burnout [7,53]. Additionally, foreign cabin crew in international airlines often encounter the acculturation stress, which possibly enhances their depression and will lead to intention to leave the organization [3,4,6].

Third, the study results confirmed that crew members’ job performance competencies regarding their respective duties (cabin service operating, galley operating, duty free sales, and safety duties during emergency situations, with the exception of safety duties during normal operations) appeared to be at different levels depending on their personalities. Despite there having been many past studies corroborating that personality types were not a strong predictor of job performance and some work-related behaviors [38,74,75], this research did yield significant correlations between personality types and occupational competencies. This study’s results support the fact that all individuals possess distinctive innate personalities, and it affect to their competency to perform certain duties in the workplace, which was consistent with findings from other studies [15,16,17].

Fourth, the present study found out Extroversion personality has higher level of stress coping competency, which were consistent with the result of previous studies [47,48].

Fifth, the earlier study regarding cabin crew’s stress- and work-related behaviors [3] found that the cabin crew without overseas experience tended to perceive more acculturation stress while they stayed in the Middle Eastern countries than those who had lived in other countries before joining airlines. In line with previous studies, this study supported that cabin crew members with overseas experience tended to have a high level of cross-cultural adjustment competency, which even influenced lessening turnover intention.

Through this result, the personality of cabin crew was generally proven to have a significant correlation with cultural adaptation, job performance or work-related competency, and stress coping. This implies that although all crew members conduct the same duties during the flight, and seem to go through some difficulties when adapting to other cultural environment, their cultural adjustment level can be vary, and even performance in each duty can appear different depending on their own personality, which also affects their mental health and turnover intention.

## 6. Implication

The academic implications of this research are as follows. First, there have been numerous studies of airline crew, yet the majority of existing studies have focused on such factors as job satisfaction, stress, burnout, and safety behavior [2,3,4,5,6,7,43,65,76,77]. This study sought to emphasize the importance of personality traits as the factor influencing cabin crew members’ cross-cultural adjustment competency, occupational competency, and coping competency. Thus, this study is the first attempt to discover international airlines’ cabin crew personality profiles using the MBTI. In addition, the findings supplement earlier MBTI studies, which failed to provide empirical support regarding the relationship between MBTI traits and individual competency.

Second, in this study, cross-cultural adjustment competency and occupational competency in respective duties were modelled by comprehensively reviewing the individual capabilities of the cabin crew member based on an earlier study. This study makes a theoretical contribution by broadening the scope of research on competency to include job performance competency, as well as the cultural adjustment of cabin crew residing overseas.

The practical implications of this study are as follows. First, the study results presented MBTI distinctive personality profiles of cabin crew in Middle Eastern airlines. It can provide useful career plan guidelines for aspiring cabin crew members, and even for former or present employees. Moreover, it suggests that each cabin crew member has unique and different personalities; thus, airlines should establish a communication training program to help them understand each other, and to avoid any conflicts within the team. Second, for cabin crew in Middle Eastern airlines, being expatriates, effective adaptation to the local community is a particularly important psychological issue. Therefore, this study gives practical suggestion to international airline companies hiring foreigners in that it is necessary to support employees by providing regular acculturation training and psychological counseling.

Second, the study highlights that crew members’ job performance competencies regarding their respective duties (cabin service operating, galley operating, duty free sales, safety duties during normal operation, and safety duties during emergency situations) appeared differently depending on their personalities. In certain airlines, each crew member, except for the onboard leaders, is assigned to serve as either a cabin service operator or galley operator on every flight. These duty positions are normally decided by a purser or crew scheduling system regardless of employees’ capacities or preferences, whereas certain airlines allow the crew members to decide their positions themselves. Our results indicate that each crew member has an inherent, distinctive personality and that it is essential to place an employee with the right talent in the right position for effective crew resource management. This is thus a practical contribution to human resource management guidelines for cabin crew recruitment, as well as for crew position assignment.

Third, this study was conducted to understand how cross-cultural adjustment competency, occupational competency, and coping competency can affect the cabin crew’s mental health and turnover intention. The results provide strategic suggestions for the airline industry to manage employees in order to maintain positive mental health conditions, improve job satisfaction, and reduce the resignation rate.

## 7. Limitation and Recommendation for Future Research

Despite the theoretical and practical implications of this research, it has several inherent limitations. First, there was a limit to collecting sufficient surveys due to the redundancies caused by COVID-19 and the time difference between Korea and the Middle Eastern countries. Moreover, it should be noted that all study participants were Korean, so these findings cannot be generalized. Therefore, for the generalization of future studies, it will be necessary to ensure that sufficient samples are collected from the Middle Eastern airlines, or we expect future relevant research to extend our model with cross-culturally generalizable samples in order to evaluate the comparison of personality profiles and their association with variables more objectively. Second, to assess MBTI personality types correctly, it is important to hold an in-depth conversational interview between MBTI experts and participants. However, due to the COVID-19 pandemic, we were forced to use online methods of data collection. Moreover, the survey questionnaire was assessed by self-reporting, which could be affect by response bias, social desirability, or subjective judgement towards the participants’ competency. Therefore, for future research regarding MBTI personality, it is recommended to carry out research using face-to-face in-depth interviews or qualitative methods.

## 8. Conclusions

Since cabin crew are essential front-line employees in the aviation industry, their duty performance and competency are crucial to increase service quality onboard. This study focused on cabin crew’s personality as variable influencing their individual competency, and enhanced Myers–Briggs Personality Indicator theory by identifying predictor variables that best predict cross-cultural adjustment competency, occupational competency, and stress coping. Additionally, the research findings provided the evidence that individuals’ different levels of cultural adjustment competency, occupational competency, and stress coping can significantly influence their mental health and turnover intention, paving the way for a broader research area.

## Figures and Tables

**Table 1:** Distribution of MBTI personality types among the respondents.

MBTI Dichotomy	Frequency	Percent	Individual Traits	Frequency	Percent
ENFP	26	15.1	Extroversion (E)	114	66.3
ESTJ	24	14.0			
ESFJ	18	10.5	Introversion (I)	58	33.7
ISTJ	16	9.3			
ESFP	15	8.7	Sensing (S)	113	65.7
ESTP	14	8.1			
ENFJ	11	6.4	Intuition (N)	59	34.3
ISFJ	10	5.8			
ISTP	8	4.7	Thinking (T)	75	43.6
ISFP	8	4.7			
INFJ	6	3.5	Feeling (F)	97	56.4
INFP	4	2.3			
ENTJ	4	2.3	Judging (J)	88	51.2
INTP	3	1.7			
ENTP	3	1.7	Perceiving (P)	84	48.8
INTJ	2	1.2			
Total	172	100.0	Total	172	100.0

**Table 2:** *t*-test for cross-cultural adjustment competency, occupational competency, and coping competency.

Variables	Frequency	Mean	SD	*t*-Value	*p*-Value	Cohen’s *d*
Cross-cultural Adjustment Competency						
Extroversion (E)	114	4.11	0.57	9.357 ***	0.000	0.713
Introversion (I)	58	3.03	0.79			
Sensing (S)	113	3.60	0.85	−3.214 **	0.002	0.245
Intuition (N)	59	4.02	0.72			
Thinking (T)	75	3.67	0.90	−1.006	0.316	0.077
Feeling (F)	97	3.80	0.77			
Judging (J)	88	3.61	0.84	−2.182 *	0.030	0.166
Perceiving (P)	84	3.89	0.79			
Occupational Competency (Cabin Service Operating)						
Extroversion (E)	114	4.31	0.50	10.765 ***	0.000	0.821
Introversion (I)	58	3.05	0.82			
Sensing (S)	113	3.78	0.86	−2.098 *	0.037	0.160
Intuition (N)	59	4.07	0.85			
Thinking (T)	75	3.58	0.93	−4.084 ***	0.000	0.311
Feeling (F)	97	4.12	0.73			
Judging (J)	88	3.81	0.91	−1.135	0.258	0.087
Perceiving (P)	84	3.96	0.82			
Occupational Competency (Galley Operating)						
Extroversion (E)	114	4.05	0.83	−1.094	0.275	0.083
Introversion (I)	58	4.20	0.81			
Sensing (S)	113	4.23	0.76	2.728 **	0.008	0.208
Intuition (N)	59	3.85	0.90			
Thinking (T)	75	4.32	0.68	3.348 **	0.001	0.255
Feeling (F)	97	3.92	0.89			
Judging (J)	88	4.32	0.69	3.742 ***	0.000	0.285
Perceiving (P)	84	3.87	0.89			
Occupational Competency (Duty Free Sales)						
Extroversion (E)	114	3.77	0.80	7.868 ***	0.000	0.600
Introversion (I)	58	2.71	0.90			
Sensing (S)	113	3.27	0.98	−2.757 **	0.006	0.210
Intuition (N)	59	3.69	0.91			
Thinking (T)	75	3.24	1.01	−2.057 *	0.041	0.157
Feeling (F)	97	3.55	0.93			
Judging (J)	88	3.40	1.05	−0.208	0.835	0.016
Perceiving (P)	84	3.43	0.90			
Occupational Competency (Safety Duties for Normal Operations)						
Extroversion (E)	114	4.01	0.70	1.075	0.284	0.082
Introversion (I)	58	3.89	0.68			
Sensing (S)	113	4.02	0.67	1.297	0.196	0.099
Intuition (N)	59	3.88	0.74			
Thinking (T)	75	4.04	0.78	1.144	0.255	0.087
Feeling (F)	97	3.92	0.63			
Judging (J)	88	4.04	0.68	1.244	0.215	0.095
Perceiving (P)	84	3.90	0.72			
Occupational Competency (Safety Duties for Abnormal and Emergency Situations)						
Extroversion (E)	114	3.93	0.68	4.852 ***	0.000	0.370
Introversion (I)	58	3.32	0.83			
Sensing (S)	113	3.76	0.78	0.656	0.513	0.050
Intuition (N)	59	3.67	0.80			
Thinking (T)	75	3.96	0.72	3.615 ***	0.000	0.276
Feeling (F)	97	3.54	0.79			
Judging (J)	88	3.73	0.78	0.074	0.941	0.006
Perceiving (P)	84	3.72	0.80			
Coping Competency						
Extroversion (E)	114	3.88	0.86	3.923 ***	0.000	0.299
Introversion (I)	58	3.31	0.97			
Sensing (S)	113	3.62	0.98	−1.240	0.217	0.095
Intuition (N)	59	3.81	0.83			
Thinking (T)	75	3.75	0.92	0.803	0.423	0.061
Feeling (F)	97	3.64	0.94			
Judging (J)	88	3.62	0.98	−0.987	0.325	0.075
Perceiving (P)	84	3.76	0.88			

* *p* < 0.05, ** *p* < 0.01, *** *p* < 0.001.

**Table 3:** Correlation analysis of cross-cultural adjustment competency, occupational competency, coping competency, mental health, and turnover intention.

Categorization	1	2	2.1	2.2	2.3	2.4	2.5	3	4	5
1. CAC	1									
2. OC	0.439 ***	1								
2.1. CS	0.549 ***	0.586 ***	1							
2.2. GO	−0.106	0.457 ***	−0.181 *	1						
2.3. DS	0.437 ***	0.647 ***	0.555 ***	−0.130	1					
2.4. SN	0.126	0.700 ***	0.134	0.462 ***	0.190 *	1				
2.5. SA	0.342 ***	0.720 ***	0.277 ***	0.351 ***	0.307 ***	0.380 ***	1			
3. CC	0.455 ***	0.235 **	0.202 **	−0.066	0.275 ***	0.054	0.257 ***	1		
4. MH	0.379 ***	0.130	0.123	−0.030	0.124	0.069	0.112	0.540 ***	1	
5. TI	−0.390 ***	−0.302 ***	−0.271 ***	0.083	−0.249 **	−0.263 **	−0.217 **	−0.392 ***	−0.524 ***	1

* *p* < 0.05, ** *p* < 0.01, *** *p* < 0.001. Note. CA = cross-cultural adjustment competency, OC = occupational competency, CS = cabin service operating, GO = galley operating, DS = duty free sales, SN = safety duty during normal operation, SA = safety duty during abnormal operation, CC = coping competency, MH = mental health, TI = turnover intention.

**Table 4:** Effects of cross-cultural competency, occupational competency, and coping competency on mental health and turnover intention, and the mediating effect of mental health.

Dependent Variables	Independent Variables	B	SE	β	*t*	*p*	F(*p*)	*R^2^*
MH	constant	4.437	0.463				26.025 (0.000)	0.317
	CAC	0.218	0.086	0.196	2.529 *	0.012		
	OC	−0.118	0.128	−0.065	−0.921	0.358		
	CC	0.460	0.071	0.466	6.504 ***	0.000		
TI	constant	6.941	0.674				16.567 (0.000)	0.228
	CAC	−0.310	0.125	−0.204	−2.480 *	0.014		
	OC	−0.369	0.186	−0.150	−1.985 *	0.049		
	CC	−0.356	0.103	−0.264	−3.463 **	0.001		
TI	constant	4.416	0.773				22.153 (0.000)	0.347
	CAC	−0.186	0.118	−0.123	−1.584	0.115		
	OC	−0.436	0.172	−0.177	−2.535 *	0.012		
	CC	−0.094	0.106	−0.070	−0.887	0.376		
	MH	−0.569	0.103	−0.416	−5.500 ***	0.000		

* *p* < 0.05, ** *p* < 0.01, *** *p* < 0.001. Note. MH = mental health, TI = turnover intention, CAC = cross-cultural adjustment competency, OC = occupational competency, CC = coping competency.

**Table 5:** Moderating effect of overseas experience.

Dependent Variables	Independent Variables	No Overseas Experience				Have Overseas Experience			
		B	β	*t*	*p*	B	β	*t*	*p*
TI	(constant)	8.724				6.253		7.405	0.000
	CAC	0.082	0.056	0.379	0.707	−0.407	−0.256	−2.753 **	0.007
	OC	−0.909	−0.445	−3.229 **	0.003	−0.171	−0.065	−0.741	0.460
	CC	−0.649	−0.483	−3.741 **	0.001	−0.285	−0.211	−2.323 *	0.022
F(*p*)		11.726 (0.000)				9.972 (0.000)			
*R* ^2^		0.481				0.192			

* *p* < 0.05, ** *p* < 0.01. Note. TI = turnover intention, CAC = cross-cultural adjustment competency, OC = occupational competency, CC = coping competency.

**Table 6:** Hypothesis testing.

Hypotheses	Links	Differences/ Standardized Estimate	Result
H1	MBTI → Cross-Cultural Adjustment Competency	E > I, S < N, J < P	Supported
H2	MBTI → Occupational Competency	Cabin: E > I, S < N, T < F Galley: S > N, T > F, J > P Duty Free: E > I, S < N, T < F Safety Normal: no difference Safety Emergency: E < I, T > F	Supported
H3	MBTI → Coping Competency	E > I	Supported
H4	Cross-Cultural Adjustment Competency → Mental Health	0.196	Supported
H5	Occupational Competency → Mental Health	−0.065	Not supported
H6	Coping Competency → Mental Health	0.466	Supported
H7	Cross-Cultural Adjustment Competency → Turnover Intention	−0.204	Supported
H7a	Moderating Effect of Overseas Experience Between Cross-Cultural Adjustment Competency and Turnover Intention.	overseas experience (0.056) no overseas experience (−0.265)	Supported
H8	Occupational Competency → Turnover Intention	−0.150	Supported
H9	Coping Competency → Turnover Intention	−0.264	Supported
H10	Mental Health → Turnover Intention	−0.416	Supported

## Data Availability

Not applicable.
